# Association between Lifestyle Changes and at-Home Hours during and after the State of Emergency Due to the COVID-19 Pandemic in Japan

**DOI:** 10.3390/nu13082698

**Published:** 2021-08-04

**Authors:** Chiharu Nishijima, Naoko Miyagawa, Nobuyo Tsuboyama-Kasaoka, Tsuyoshi Chiba, Motohiko Miyachi

**Affiliations:** 1 Department of Food Function and Labeling, National Institute of Health and Nutrition, National Institutes of Biomedical Innovation, Health and Nutrition, 1-23-1 Toyama, Shinjuku-ku, Tokyo 162-8636, Japan; c-nishijima@nibiohn.go.jp; 2 International Center for Nutrition and Information, National Institute of Health and Nutrition, National Institutes of Biomedical Innovation, Health and Nutrition, 1-23-1 Toyama, Shinjuku-ku, Tokyo 162-8636, Japan; nmiya@nibiohn.go.jp (N.M.); ntsubo@nibiohn.go.jp (N.T.-K.); 3 Department of Physical Activity Research, National Institute of Health and Nutrition, National Institutes of Biomedical Innovation, Health and Nutrition, 1-23-1 Toyama, Shinjuku-ku, Tokyo 162-8636, Japan; miyachi@nibiohn.go.jp

**Keywords:** physical activity, sleep duration, eating behaviors, body weight, coronavirus disease

## Abstract

Lifestyle changes during the coronavirus disease (COVID-19) lockdown have been previously examined, but there is limited understanding about changes after such restrictions were lifted. This study examines changes in lifestyle habits and body weight among the Japanese population with regard to the length of at-home hours both during (April to May) and after (September) the nationwide stay-at-home request compared to those before the COVID-19 pandemic (January 2020). An online survey was conducted in September 2020 involving 10,000 Japanese survey monitors, selected according to population distribution. During the stay-at-home request, 34% participants extended their at-home hours. More respondents in the group with extended at-home hours experienced an increase or decrease in total physical activity, snacking, food intake, alcohol drinking, and body weight than those in the group with nonextended at-home hours. Some of these changes had a trend according to age. The prevalence of most of these changes decreased when at-home hours returned to normal after the stay-at-home request period; however, increased alcohol consumption and increased or decreased body weight persisted. Our findings suggest that close monitoring for further health outcomes and age-appropriate measures to encourage favorable health behaviors is needed.

## 1. Introduction

The coronavirus disease (COVID-19) pandemic is an urgent global public health threat. The spread of COVID-19 has resulted in closing or restricting borders in many countries, with one-third of the global population placed under some social restrictions [1]. National and regional lockdowns implemented in other countries have reduced the rate of COVID-19 transmission, but have pushed people to stay at home. Prolonged at-home hours brought alterations in lifestyle behaviors such as reduced physical activity (PA) and the increased consumption of unhealthy foods, together with difficulty in maintaining body weight [2,3,4]. These unfavorable changes may have adverse metabolic effects [5], and in turn, may increase the risk of diabetes, cardiovascular disease, or more serious health outcomes in the long term.

In late February 2020, an increase in the number of infected people was observed in Japan. With the continuous spread of the infection, the first nationwide declaration of a state of emergency was issued on 16 April 2020. Following the declaration, the public was asked to refrain from nonessential activities outside the home until the state of emergency was lifted on 26 May 2020. All commercial facilities were invited to close or shorten their business hours, and companies were advised to encourage remote working. The stay-at-home request between April and May 2020 in Japan was not compulsory as lockdowns implemented in other countries were, but the number of people transiting through major train stations and downtown areas of the country decreased to approximately 54% of the previous volume [6], suggesting that many people stayed at home. After the state of emergency had been lifted, restrictions in public behavior greatly loosened with the adoption of public-health measures such as wearing masks, handwashing, and avoiding the 3Cs (closed spaces with poor ventilation, crowded spaces with many people nearby, and close-contact settings such as close-range conversations). Theaters, department stores, restaurants, and sports clubs resumed operations, and government-based travel campaigns were also promoted to restore the economy. The second wave of COVID-19 infections occurred in late July, two months after the declaration had been lifted. However, the spread of the infection was mitigated by a request to reduce the operating hours of restaurants, a repeated call for the public to practice basic public-health measures, and the voluntary restraint of the public, without declaring a state of emergency [7].

Evidence indicates that the state of emergency between April and May 2020 caused a decrease in PA among workers [8] and a deterioration in general health status due to significant changes in lifestyle and body weight among urban residents [9]. However, these changes were observed in groups that were susceptible to the stay-at-home request. These groups were the most affected by the extension of at-home hours, and the effects of the nationwide stay-at-home request remain unclear. Furthermore, people’s lifestyle may have changed further after the lifting of restrictions. Tracking changes in livelihoods during and after the stay-at-home request period is important for considering appropriate public-health measures aimed at the public-health impact of COVID-19.

The purpose of this study is to elucidate the nationwide impact of the stay-at-home request with regard to extended at-home hours by clarifying changes in lifestyle habits and body weight during and after the stay-at-home request period. We conducted an online survey of respondents following the same sex, age, and regional distribution of Japanese citizens in September 2020, and examined the situation or changes before the COVID-19 pandemic (around January 2020), during the stay-at-home request period (April to May 2020), and after the stay-at-home request period (September 2020).

## 2. Materials and Methods

### 2.1. Online Survey and Study Population

This cross-sectional survey was conducted by Rakuten Insight (Tokyo, Japan), an online research company, between 9 and 14 September 2020. Rakuten Insight has 2.2 million nationwide monitors. The Rakuten Group monitors fraudulent registrants such as spoofing registrations and duplicate registrations by sharing the basic registration information of members. Furthermore, as part of their quality control of survey results, Rakuten Insight introduced an automatic computer-based check system and a visual-quality control system for unacceptable responses such as inconsistent or fraudulent answers for the obtained responses. Survey participants are provided with e-points that can be used within the Rakuten Group.

The subjects in this study were adults aged ≥ 20 years who had not been infected by COVID-19 at the time of survey. The survey cohort comprised 10,000 people on the basis of area of residence, sex, and age distribution obtained from the 2015 Population Census [10]. An email with a survey cooperation request and a webpage link to the survey was sent to 93,925 computer-randomized monitors. An explanation of this study was provided at the beginning of the survey page, and only those individuals who had agreed to participate answered the questionnaire. To avoid missing data, questions were given one per page and participants had to answer any to go forward. Complete responses were collected on a first come, first served basis, until the numbers reached the quotas for areas of residence, sex, and age in accordance with the proportions as reported in the 2015 Population Census. A total of 12,117 (12.9%) responses were obtained. After excluding unacceptable responses, a total of 10,000 respondents were randomly selected from those with complete data and delivered to us from the research company. The response data were given an ID unique to that survey and did not include the individual’s name or contact information. This study was performed in accordance with the Declaration of Helsinki, with approval from the Ethical Review Board of the National Institutes of Biomedical Innovation, Health, and Nutrition (KENEI-134; approval date: 4 August 2020).

### 2.2. Questionnaire

The questionnaire in this study included sociodemographic variables, at-home hours, PA, anxiety about COVID-19 infection, sleep duration, dietary habits, lifestyle habits, and body weight. Participants were asked for details at three time points: before the COVID-19 pandemic (around January 2020), during the stay-at-home request period (April to May 2020), and after the stay-at-home request period (September 2020). Thus, they were asked to recall details from before the COVID-19 pandemic and during the stay-at-home request, as well as to report their current situation after the stay-at-home request lifted.

The average at-home hours on working days for working people or weekdays for non-working people such as students and homemakers were reported by choosing one of the following time frames: 0–8, 8–12, 12–16, 16–20, or 20–24 h. The mean at-home hours were calculated using the median value for each time frame. For PA, the Japan Public Health Center Physical Activity Questionnaire short form used in the Japan Public Health Center-based prospective study was used in this study [11]. This questionnaire asks the length of time spent on heavy physical work or strenuous exercise, walking and standing, and sedentary activity. The amount of total PA was calculated by the length of time spent on the respective activity multiplied by the assigned metabolic equivalents. For anxiety about COVID-19 infection, participants were asked to rate their level of worry about catching COVID-19 during and after the stay-at-home request period with response options of “very anxious”, “a little anxious”, “not sure”, “not too anxious”, and “not anxious”. For sleep duration, the average sleep duration on weekdays or working days was selected from six time frames [12]. For dietary habits, participants were asked about the frequency of the following: eating breakfast, having a well-balanced diet, and the habit of eating three meals at a given time a day. A well-balanced diet was defined as a meal consisting of a staple (rice, bread, and noodle dishes), main dish (dishes mainly consisting of fish, meat, eggs, soybeans, and soy products), and side dish (vegetables, seaweed, and mushrooms) eaten at least twice a day [12]. Response options for dietary habits were “almost every day”, “4 to 5 days a week”, “2 to 3 days a week”, and “rarely”. Changes in PA, sleep duration, and dietary habits during and after the stay-at-home request period were determined by comparing the length of time or frequency to that before the COVID-19 pandemic.

For changes in lifestyle habits and food intake, the questions included changes in waketime, bedtime, the frequency of defecation, smoking, drinking, main meals, snacking, and the consumption of foods such as cereals, potatoes, soy and soy products, vegetables, fruits, seaweed, fish and seafood, meat, eggs and egg products, milk and milk products, oily foods, salty snacks and confectionary, and sugar-sweetened beverages. Participants were asked to select items based on whether changes “increased” or “decreased”, or “became earlier” or “became later”. Items that were not selected were considered to be “unchanged”. For changes in body weight, participants self-reported body weight before the COVID-19 pandemic as an integer and changes in body weight during and after the stay-at-home request period using the 11 following options: no change, gained/lost 1 kg, gained/lost 2 kg, gained/lost 3 kg, gained/lost 4 kg, and gained/lost ≥5 kg. For sociodemographic data, participants’ age, sex, education, annual household income, and occupation were collected.

### 2.3. Statistical Analysis

To examine the longitudinal impact of extended at-home hours, participants were divided into groups according to the change in at-home hours during the stay-at-home request period by comparing the median values of at-home hours before the COVID19 pandemic and during the stay-at-home request period. Participants with “increased” at-home hours were included in the group with “extended at-home hours” (*n* = 3272), while those with “unchanged” at-home hours were included in the group with “nonextended at-home hours” (*n* = 6373). Participants with “decreased” at-home hours (*n* = 355) were excluded from analysis after confirming that their exclusion did not affect sociodemographic attributes, because they deviated from the purpose of the study. Therefore, 9645 people were included in the analysis.

All data yielded descriptive statistics, and differences in the distribution between groups were compared using the chi-squared test. For the effect size of the chi-squared test, Cramer’s V was used with values between 0 and 1, where 1 indicates the strongest association. The proportion of participants who consumed alcohol and smoked cigarettes was calculated among alcohol drinkers (*n* = 4220) and smokers (*n* = 1747). R version 4.1.0 (R Core Team, 2021, Vienna, Austria) was used for statistical analysis, and a *p* value of <5% was considered to be statistically significant.

## 3. Results

### 3.1. Characteristics

Table 1 shows the characteristics of participants. During the stay-at-home request period, 3272 (33.9%) people extended their at-home hours (Extended), and the average at-home hours were 18.4 ± 4.1, which was 6.3 h longer than that of people with nonextended at-home hours (Nonextended, 12.1 ± 6.7 h). More participants living in metropolitan areas had extended at-home hours than those in other areas. More participants with a higher educational background and higher annual household income, clerical workers, technicians and associate professionals, and students were in the Extended group.

### 3.2. Lifestyle and Dietary Habits during and after the Stay-at-Home Request Period

#### 3.2.1. Physical Activity

During the stay-at-home request period, the proportion of participants who spent ≥3 h/d walking and standing was lower in the extended group than that in the nonextended group (Table 2). The proportion of participants who had ≥8 h/day of sedentary activity was two times higher among people in the Extended group than among those in the Nonextended group. Regarding PA changes compared to before the COVID-19 pandemic, approximately 40% of participants in the Extended group experienced decreased walking and standing and increased sedentary activity during the stay-at-home request period; almost half of people in the same group had decreased total PA in that period. Overall, the proportions of participants who reported an increase or decrease in all items were higher in the Extended group than those in the Nonextended group, both during and after the stay-at-home request period (all, *p* < 0.001). However, the effect size of the association in all items diminished after the stay-at-home request period.

#### 3.2.2. Anxiety about COVID-19 Infection and Sleep Habits

Table 3 shows the distribution of anxiety about COVID-19 infection and sleep duration, and changes in sleep duration, waketime, and bedtime compared to those before the COVID-19 pandemic. During the stay-at-home request period, most people felt anxious about COVID-19 infection regardless of whether at-home hours were extended. In terms of sleep duration, the prevalence of a short sleep duration (<5 h) during the stay-at-home request period was less than a half of that in the Nonextended group (11.1%) than that in the Extended group (4.8%). This proportion remained low at 5.9% even after the stay-at-home request period. Compared to before the COVID-19 pandemic, 30.5% of participants in the Extended group reported a longer sleep duration during the stay-at-home request period; however, this proportion was reduced to 18.4% after the period. The proportions of people who changed sleep habits (became longer or shorter, or later or earlier) in all items were higher in the Extended group than those in the Nonextended group, both during and after the stay-at-home request period (all, *p* < 0.001).

#### 3.2.3. Lifestyle and Dietary Habits

Compared to before the COVID-19 pandemic, during the stay-at-home request period, the number of people reporting an increase in alcohol consumption (21.1%) and the number of those reporting a decrease in alcohol consumption (13.4%) were higher in the Extended group than those in the Nonextended group. This proportion remained high even after the stay-at-home request period (Table 4). The proportion of participants reporting an increase in the frequency of main meals and snacking in the Extended group (10.7% and 31.1%, respectively) was three times higher than that in the Nonextended group. This proportion decreased by about half after the stay-at-home request period. With regard to dietary habits, participants reporting an increase in a “well-balanced diet” and the “habit of eating three meals at a given time a day” during the stay-at-home period was five times higher among participants in the Extended group than among those in the Nonextended group. Changes in food intake during and after the stay-at-home request period compared to those before the COVID-19 pandemic are shown in Table S1. In all foods, the proportion of participants reporting an increase or decrease during and after the stay-at-home request period was higher in the Extended group than that in the Nonextended group. More than 25% of participants in the Extended group reported increased consumption of cereals, salty snacks, and confectionary during the stay-at-home request period.

### 3.3. Changes in Body Weight during and after Stay-at-Home Request Period

In self-reported changes in body weight compared to that before the COVID-19 pandemic, weight gain was observed in 39.9% of participants in the Extended group during the stay-at-home request period (Table 5). Of these, 25.9% reported an increase in weight by 1–2 kg. The proportion of participants who had gained weight remained at 35.3% even after the stay-at-home request period. At the opposite end of the spectrum, 13.9% of participants reported weight loss during the stay-at-home request period. This proportion slightly increased after the stay-at-home request period to 20.7% among participants with Extended at-home hours.

### 3.4. Lifestyle Habits and Body Weight Changes according to Age Group during and after Stay-at-Home Request Period in the Extended Group

Lifestyle habits and body weight changes among participants in the Extended group (*n* = 3272) compared to those before the COVID-19 pandemic are presented according to age group (Table S2). Total PA decreased in approximately 50% of the participants (during the stay-at-home request period) or approximately 30% of the participants (after the stay-at-home request period) across all age groups, and was the highest among those aged ≥ 60 years. Conversely, approximately 15% of respondents in their 20 s showed an increase in total PA and heavy physical work or strenuous exercise. The proportion of respondents in their 20 s and ≥60 s with increased sedentary activity was high during and after the stay-at-home request period. The proportion of participants who reported changes in the sleep duration, waketime, and bedtime, the amount of drinking and smoking, frequencies of defecation, main meals, and snacking was higher in the younger age groups than that in the older age groups. Conversely, there were no certain trends in increased body weight according to age group. The proportion of participants who had lost weight was higher in the younger age groups than that in the older age groups at both time points: 22.0% versus 11.0% (during the stay-at-home request period) and 31.1% versus 16.9% (after the stay-at-home request period).

### 3.5. Distribution of Body Mass Index before the COVID-19 Pandemic, and during and after Stay-at-Home Request Period in the Extended Group

**Figure 1:**
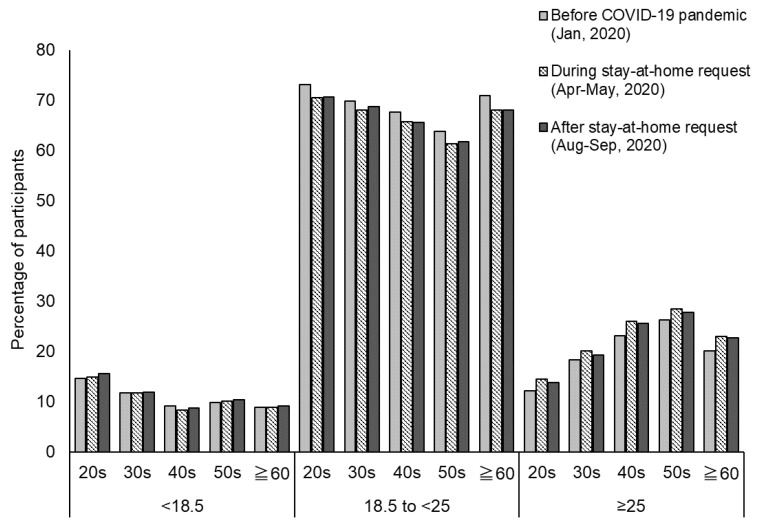
Distribution of body mass index before the COVID-19 pandemic, during the stay-at-home request period, and after the stay-at-home request period among participants with extended at-home hours during the stay-at-home request period (*n* = 3272).

Among participants in the Extended group (*n* = 3272), the proportion of people with a BMI of ≥25 increased during the stay-at-home request period (Figure 1); the proportion tended to decrease after the stay-at-home request period, but did not return to pre-COVID-19 pandemic levels. There was a slight increase in the number of underweight persons among respondents in their 20 s and 50 s during and after the stay-at-home request period.

### 3.6. Changes in at-Home Hours, and Lifestyle and Body Weight Changes after Stay-at-Home Request Period in the Extended Group

Among people in the Extended group (*n* = 3272), there were 1328 (40.6%) people with at-home hours returned to or reduced to pre-COVID-19 pandemic levels after the stay-at-home request period (Table 6), the majority of which (1944 (59.4%)) still had extended at-home hours. After the stay-at-home request period, among people whose at-home hours returned to or reduced to pre-COVID-19 pandemic levels, lifestyle habits (heavy physical work or strenuous exercise, walking and standing, sedentary activity, sleep duration, frequency of defecation, main meal, and snacking) were unchanged compared to those before the COVID-19 pandemic in more than 80% of participants; however, the percentage of those who had unchanged body weight was only 47.6%, which was comparable to the proportion of people with extended at-home hours (41.5%). Furthermore, the proportion of participants with increased alcohol consumption and smoking habit among people whose at-home hours returned to or reduced to the pre-COVID-19 pandemic levels remained at approximately 15%.

## 4. Discussion

This study examined the association between the extension of at-home hours, and changes in lifestyle habits and body weight as an effect of the stay-at-home request due to the declaration of the state of emergency during the COVID-19 pandemic. In total, 33.9% of the total participants had extended at-home hours due to the stay-at-home request. This proportion is close to the remote-work implementation rate (28%) after the declaration of a state of emergency in Japan [13]. In September 2020, four months after the stay-at-home request, we assumed that people’s lives were returning to normal because people were returning to urban areas and tourist spots. However, our study revealed that only 40.6% of respondents with extended at-home hours returned to normal at-home hours. Furthermore, we observed both an increase and decrease in PA, diet, and body weight among people with extended at-home hours, suggesting the possibility of a polarization of effects.

Reduced activity was reported as a global trend during lockdown [4,14]. In this study, the decrease in PA during the stay-at-home request period was observed in 25.3% of all participants and 49.2% of participants with extended at-home hours. In other countries, reduced PA was reported in 43–63% of persons [2]. This indicates that the overall proportion of persons with decreased PA among our participants was relatively low. However, when limited to participants with extended at-home hours, our results were similar to those of studies conducted in Australia (43%), the United States (43%), Poland (43%), and China (48%), where lockdown conditions were investigated [15,16,17]. In our study, the decrease in PA differed by age group. While walking and standing decreased across all age groups, heavy physical work or strenuous exercise decreased or increased among some younger adults. An increase in media exposure after the COVID-19 pandemic was reported [9], and exercise and fitness promotions were introduced on multiple media [18]. Furthermore, fitness videos and social interactions motivate followers that are already physically active [19]. On this basis, participants with increased heavy physical work or strenuous exercise in this study sought to have some opportunity to exercise, such as via a home-based exercise program.

Previous studies reported increased snacking and main meals, and both favorable and unfavorable dietary changes during lockdown [15,17,20,21,22,23]. In this study, regarding favorable changes, the frequency of breakfast, a well-balanced diet, and the habit of eating three meals at a given time a day increased, and these changes led to an increase in main-meal frequency. Participants with extended at-home hours reported an increase in vegetable, fruit, and dairy-product consumption, which are low in poor-quality diets [24]. Previous studies showed that favorable dietary changes are associated with previously poor everyday dietary quality [21], and staying at home may have resulted in dietary improvement among individuals with poor-quality diets. A healthy diet with more vegetables and fruits is also associated with an increased intake of home-cooked food [17], and an increase in home-cooked food was observed during lockdown [15,21,22]. Therefore, favorable dietary changes may have occurred due to increased at-home hours, which allowed for more time for cooking and eating at home [22,25]. There was also a favorable change in the prevalence of short sleepers being reduced among participants with extended at-home hours. This implies that the extension at-home hours brought time to spare for longer sleep and to have breakfast. However, for individuals whose at-home hours returned to normal after the stay-at-home request, their dietary habits, food consumption, and sleep duration returned to normal. Therefore, if this temporal factor is removed, favorable dietary changes and sleep duration may not persist and may be difficult to establish.

In terms of unfavorable dietary changes, as observed in various countries during lockdown [17,20,21,22,23,26], increased snacking frequency and increased consumption of alcohol, salty snacks and confectionery, oily foods, and sugar-sweetened beverages were observed among individuals with extended at-home hours during the stay-at-home request. For individuals whose at-home hours returned to normal after the stay-at-home request period, the proportion of people with unfavorable changes decreased. This result is consistent with that reported by a recent study by Bhutani et al. comparing changes in behaviors during peak and post-lockdown [27]. The increase in unhealthy food consumption during lockdown was associated with changes in mood including lack of motivation, anxiety, and boredom [2]. The aforementioned research team also reported that participants experienced less boredom and more control over cravings after lockdown [27]. On the basis of these findings, after the stay-at-home request, PA could have increased due to the increase in permissible outdoor activities, and it may be that the increase in the consumption of snacks was suppressed when stress and boredom were alleviated. However, with regard to alcohol consumption, which is also reported to have increased due to stress and boredom [21,26], even after the stay-at-home request period, the proportion of individuals with increased alcohol consumption did not decrease, particularly among relatively young age groups. This may need close monitoring for the longer-term impact of increased alcohol intake.

Among participants with at-home hours that returned to normal, the proportion of those who gained or lost weight did not decrease, and the rate of obesity and underweight presentation increased compared to that before the COVID-19 pandemic. This is consistent with the findings reported by Bhutani et al. in the United States [27], and our study also suggests that the disruption of weight management during the stay-at-home request period could persist. Difficulty in weight management is not only a risk factor for chronic disease [28], but also a risk factor for frailty in elderly people [29]. For respondents aged ≥ 60 years, increased sedentary activity was the most prominent during and after the stay-at-home request period, which is another risk factor for frailty [30]. An increase in the prevalence of being underweight among individuals in their 20 s during and after the stay-at-home request period was also observed. Phillipou et al. reported that 28% of the general population had dietary restrictions during lockdown, with weak but positive correlation between dietary restrictions and PA [31]. Although we did not examine the correlation, the proportion of individuals with both increased PA and decreased main-meal frequency was the highest among the group of participants in their 20 s. Increasing the number of underweight people, particularly among women in the reproductive age, is a serious concern [32]. Altogether, some participants still had increased alcohol intake, or had gained or lost body weight among those who returned their at-home hours normal, and may need age-appropriate intervention.

In addition, among participants with extended at-home hours that continued after the stay-at-home request period (in September), the prevalence of an unfavorably changed lifestyle such as decreased PA, extended sedentary activity, increased snacking frequency, increased drinking, and increased smoking was comparable to that observed among participants during the stay-at-home request period (in April). This implies that individuals who had extended at-home hours for almost half a year did not adopt measures to address the deterioration in their lifestyle. Although the increased sleep duration observed among participants with extended at-home hours plays a protective role in cardiovascular disease for those reporting a shorter sleep duration [33], prolonged sedentary time increases the risk of cardiovascular disease [34]. Obesity, an unhealthy diet, and smoking are also risk factors for chronic illness [28]; therefore, it is important to raise awareness about health-protective behaviors.

The results of this study need to be interpreted considering the following limitations. First, since this study was an online survey, the participants were survey monitors, which makes generalization difficult. We selected a research company with nationwide monitors at all segments of people to closely resemble national estimates. Random sampling by age, sex, and place of residence was considered; however, due to the nature of the online survey, participants may have been limited to persons with extensive media exposure at the time of the survey. Second, all responses were self-reported and may have included subjective bias. In addition, data on the “before COVID-19 pandemic” and “during stay-at-home request” periods may involve recall bias. All data obtained over time were derived from the same person, but a causal relationship could not be estimated. Third, the stay-at-home request made people not only stay at home on an individual level, but also made restaurants, gyms, public and amusement facilities, and tourist businesses close or shorten their operating hours. These situations might have caused the changes in dietary habits and physical activities found in our results. Despite these limitations, this study is the first to report longitudinal changes in lifestyle habits with regard to at-home hours.

## 5. Conclusions

This study clarified that the extension of at-home hours had positive effects such as securing sleep time and improvement of eating habits, and negative effects such as a decrease in PA, increase in snacking, and difficulty in maintaining weight. Although at-home hours returned to normal after the stay-at-home request for some people, the positive effects were not maintained, and some negative effects, including the increase in alcohol consumption and changes in body weight, did not immediately return to normal. Since the magnitude of effects and the possible longer-term impact differ by age, our findings suggest the need for age-appropriate measures to prevent a further impact on health.

## Figures and Tables

**Table 1:** Characteristics of participants according to changes in at-home hours during stay-at-home request period.

	All	Nonextended ^1^	Extended ^1^	*p* Value ^2^ (Cramer’s V ^3^)
	*n*	%	%	
Number (%)	9645	66.1	33.9	
Sex (%)				0.008
Male	4595	67.4	32.6	(0.027)
Female	5050	64.9	35.1	
Age, years (%)				0.001
20–29	1132	61.4	38.6	(0.045)
30–39	1431	65.1	34.9	
40–49	1702	68.6	31.4	
50–59	1441	68.2	31.8	
≥60	3939	65.9	34.1	
Residential area ^4^ (%)				<0.001
Metropolitan area	4024	56.3	43.7	(0.180)
Urban area	2191	69.9	30.1	
Rural area	3430	75.1	24.9	
Education (%)				<0.001
Junior high school	142	79.6	20.4	(0.142)
High school	2639	72.6	27.4	
College, vocational school	2250	68.6	31.4	
University or higher	4358	59.3	40.7	
Did not wish to answer	256	84.0	16.0	
Annual household income ^5^ (%)				<0.001
JPY < 2 million	813	69.6	30.4	(0.078)
JPY 2 to <4 million	2142	68.5	31.5	
JPY 4 to <6 million	1970	66.2	33.8	
JPY ≥ 6 million	2949	60.8	39.2	
Did not wish to answer	1771	70.1	29.9	
Occupation (%)				<0.001
Clerical workers	1927	60.5	39.5	(0.181)
Managers	712	64.6	35.4	
Service and sales workers	1088	66.8	33.2	
Craft and related trade workers	819	83.0	17.0	
Technicians and associate professionals	543	55.2	44.8	
Medical professions	629	79.2	20.8	
Homemakers	1727	63.2	36.8	
Students	133	26.3	73.7	
No occupation	2067	68.5	31.5	

^1^ Changes in at-home hours during the stay-at-home request period compared to those in the period before the COVID-19 pandemic. ^2^ Differences in distribution between groups were examined by chi-squared test. ^3^ Ranges from 0 to 1, where 1 indicates a strong association. ^4^ Areas are defined as follows: metropolitan, prefectures ≥ 8 million inhabitants and outlying prefectures; urban, prefectures 5 to <8 million inhabitants and outlying prefectures; rural, prefectures other than metropolitan and urban areas. ^5^ A currency exchange rate of JPY 100 = USD 0.90 is applicable.

**Table 2:** Physical activity (PA) during and after stay-at-home request period according to changes in at-home hours during stay-at-home request.

	During Stay-at-Home Request Period		*p* Value ^2^ (Cramer’s V ^3^)	After Stay-at-Home Request Period		*p* Value ^2^ (Cramer’s V ^3^)
	Nonextended at-Home Hours ^1^ (*n* = 6373)	Extended at-Home Hours ^1^ (*n* = 3272)		Nonextended at-Home Hours ^1^ (*n* = 6373)	Extended at-Home Hours ^1^ (*n* = 3272)	
	%	%		%	%	
Heavy physical work or strenuous exercise (/day)						
None	65.9	60.7		63.7	55.7	
<1 h	21.5	29.4		21.2	28.7	
≥1 h	12.6	9.9		15.1	15.6	
Walking and standing						
<1 h	31.0	37.1		29.6	24.5	
1 to <3 h	41.3	45.6		41.0	46.1	
≥3 h	27.7	17.4		29.4	29.3	
Sedentary activity						
<3 h	26.6	10.4		27.7	15.7	
3 to <8 h	51.5	44.9		51.3	51.4	
≥8 h	21.9	44.7		21.0	32.9	
PA changes compared to those before the COVID-19 pandemic						
Total PA			<0.001			<0.001
Decreased	13.1	49.2	(0.420)	9.8	31.0	(0.285)
No change	82.4	41.8		83.8	59.0	
Increased	4.5	9.0		6.3	10.1	
Heavy physical work or strenuous exercise			<0.001			<0.001
Decreased	8.6	23.8	(0.239)	6.3	16.1	(0.176)
No change	87.7	67.9		88.9	75.9	
Increased	3.7	8.3		4.7	8.0	
Walking and standing			<0.001			<0.001
Decreased	7.9	43.6	(0.432)	6.1	24.9	(0.276)
No change	90.0	52.3		90.8	70.4	
Increased	2.1	4.2		3.1	4.7	
Sedentary activity			<0.001			<0.001
Decreased	1.3	3.8	(0.406)	1.8	3.6	(0.266)
No change	92.5	59.2		93.4	75.1	
Increased	6.2	37.0		4.8	21.3	

^1^ Changes in at-home hours during stay-at-home request period compared to those in the period before the COVID-19 pandemic. ^2^ Differences in distribution between the groups were examined by chi-squared test. ^3^ Ranges from 0 to 1, where 1 indicates a strong association.

**Table 3:** Anxiety about COVID-19 infection and sleep habits during and after stay-at-home request period according to changes in at-home hours during the stay-at-home request period.

	During Stay-at-Home Request Period		*p* Value ^2^ (Cramer’s V ^3^)	After Stay-at-Home Request Period		*p* Value ^2^ (Cramer’s V ^3^)
	Nonextended at-Home Hours ^1^ (*n* = 6373)	Extended at-Home Hours ^1^ (*n* = 3272)		Nonextended at-Home Hours ^1^ (*n* = 6373)	Extended at-Home Hours ^1^ (*n* = 3272)	
	%	%		%	%	
Anxiety about COVID-19 infection						
Anxious ^4^	66.2	78.0		57.9	66.5	
Not sure	14.9	7.5		19.0	14.3	
Not anxious ^5^	18.9	14.5		23.1	19.2	
Sleep duration						
<5 h	11.1	4.8		11.3	5.9	
5 to <6 h	23.1	18.0		23.5	22.2	
6 to <7 h	35.1	36.1		35.5	39.2	
7 to <8 h	21.9	28.3		21.2	24.7	
8 to <9 h	6.2	10.0		6.0	6.7	
≥9 h	2.4	2.8		2.4	1.4	
Sleep habit changes compared to those before the COVID-19 pandemic						
Sleep duration			<0.001			<0.001
Became longer	3.6	30.5	(0.399)	3.4	18.4	(0.271)
No change	94.3	63.9		93.1	74.8	
Became shorter	2.1	5.6		3.5	6.8	
Waketime			<0.001			<0.001
Became later	4.3	24.9	(0.330)	3.0	13.7	(0.252)
No change	91.4	66.4		91.4	73.1	
Became earlier	4.3	8.7		5.6	13.2	
Bedtime			<0.001			<0.001
Became later	5.3	18.5	(0.278)	4.8	13.2	(0.213)
No change	88.6	65.6		89.0	72.3	
Became earlier	6.1	15.9		6.1	14.4	

^1^ Changes in at-home hours during stay-at-home request period compared to those in the period before the COVID-19 pandemic. ^2^ Differences in distribution between the groups were examined by chi-squared test. ^3^ Ranges from 0 to 1, where 1 indicates a strong association. ^4^ Sum of “very anxious” and “a little anxious”. ^5^ Sum of “not too anxious” and “not anxious”.

**Table 4:** Lifestyle and dietary habits during and after stay-at-home request period according to the changes in at-home hours during the stay-at-home request period.

	During Stay-at-Home Request Period		*p* Value ^2^ (Cramer’s V ^3^)	After Stay-at-Home Request Period		*p* Value ^2^ (Cramer’s V ^3^)
	Nonextended at-Home Hours ^1^ (*n* = 6373)	Extended at-Home Hours ^1^ (*n* = 3272)		Nonextended at-Home Hours ^1^ (*n* = 6373)	Extended at-Home Hours ^1^ (*n* = 3272)	
	%	%		%	%	
Lifestyle and dietary changes compared to those before the COVID-19 pandemic						
Amount of drinking ^4^			<0.001			<0.001
Increased	8.9	21.1	(0.242)	8.5	16.2	(0.208)
No change	86.0	65.5		85.7	68.6	
Decreased	5.1	13.4		5.8	15.2	
Amount of smoking ^5^			<0.001			<0.001
Increased	9.4	28.4	(0.310)	8.2	22.1	(0.236)
No change	87.9	61.2		87.4	67.9	
Decreased	2.8	10.4		4.4	10.1	
Frequency of defecation			<0.001			<0.001
Increased	2.4	6.8	(0.137)	2.4	5.8	(0.120)
No change	95.9	88.8		95.5	89.2	
Decreased	1.7	4.5		2.1	5.0	
Frequency of main meals			<0.001			<0.001
Increased	3.3	10.7	(0.178)	2.3	5.3	(0.124)
No change	95.3	85.0		95.6	89.1	
Decreased	1.4	4.3		2.1	5.6	
Frequency of snacking			<0.001			<0.001
Increased	10.2	31.1	(0.273)	7.4	17.8	(0.190)
No change	88.0	65.2		89.4	74.9	
Decreased	1.7	3.7		3.1	7.4	
Frequency of breakfast			<0.001			<0.001
Increased	1.4	6.2	(0.170)	1.6	5.2	(0.130)
No change	97.3	89.2		96.9	90.8	
Decreased	1.3	4.6		1.5	4.0	
Frequency of a well-balanced diet ^6^			<0.001			<0.001
Increased	2.1	9.4	(0.200)	1.9	6.6	(0.154)
No change	95.9	84.7		95.7	87.4	
Decreased	2.0	6.0		2.4	5.9	
Habit of eating three meals at a given time of the day			<0.001			<0.001
Increased	2.2	11.6	(0.248)	2.1	8.1	(0.172)
No change	95.8	80.5		95.7	86.2	
Decreased	2.0	7.9		2.2	5.7	

^1^ Changes in at-home hours during stay-at-home request period compared to those in the period before the COVID-19 pandemic. ^2^ Differences in distribution between the groups were examined by chi-squared test. ^3^ Ranges from 0 to 1, where 1 indicates a strong association. ^4^ Excluding those who never drink. ^5^ Excluding those who never smoke. ^6^ Defined as a meal consisting of a staple (rice, bread, and noodle dishes), main dish (dishes mainly consisting of fish, meat, eggs, soybeans, and soy products), and side dish (vegetables, seaweeds, and mushrooms) eaten at least twice a day.

**Table 5:** Changes in body weight during and after stay-at-home request period according to changes in at-home hours during the stay-at-home request period.

	During Stay-at-Home Request Period		*p* Value ^2^ (Cramer’s V ^3^)	After Stay-at-Home Request Period		*p* Value ^2^ (Cramer’s V ^3^)
	Nonextended at-Home Hours ^1^ (*n* = 6373)	Extended at-Home Hours ^1^ (*n* = 3272)		Nonextended at-Home Hours ^1^ (*n* = 6373)	Extended at-Home Hours ^1^ (*n* = 3272)	
	%	%		%	%	
Body weight changes compared to that before the COVID-19 pandemic						
+5 kg or more	2.0	3.8		2.4	4.2	
+4 kg	1.0	2.7		1.5	2.6	
+3 kg	3.8	7.5		4.2	6.0	
+2 kg	5.9	14.0		6.3	11.8	
+1 kg	8.1	11.9		7.6	10.7	
0 (No change)	69.4	46.2		62.9	43.9	
−1 kg	3.4	4.1		5.2	6.1	
−2 kg	2.7	4.2		3.8	6.1	
−3 kg	1.7	2.5		2.5	3.3	
−4 kg	0.5	0.9		1.1	1.8	
−5 kg or more	1.5	2.3		2.5	3.5	
Reposting			<0.001			<0.001
Increased	20.8	39.9	(0.230)	22.0	35.3	(0.183)
No change	69.4	46.2		62.9	43.9	
Decreased	9.8	13.9		15.0	20.7	

^1^ Changes in at-home hours during stay-at-home request period compared to those in the period before the COVID-19 pandemic. ^2^ Differences in distribution between groups were examined by chi-squared test. ^3^ Ranges from 0 to 1, where 1 indicates a strong association.

**Table 6:** Changes in at-home hours after stay-at-home request period among people with extended at-home hours during stay-at-home request period (*n* = 3272), and lifestyle and body weight changes after stay-at-home request period.

	After Stay-at-Home Request Period		*p* Value ^2^ (Cramer’s V ^3^)
	At-Home Hours Returned/Reduced to Pre-COVID-19 Pandemic Levels ^1^ (*n* = 1328)	At-Home Hours Continued to be Extended ^1^ (*n* = 1944)	
	%	%	
Lifestyle and body weight changes compared to before the COVID-19 pandemic			
Total PA			
Decreased	13.9	42.6	<0.001
No change	75.9	47.4	(0.313)
Increased	10.2	10.0	
Heavy physical work or strenuous exercise			
Decreased	9.0	20.9	<0.001
No change	83.3	70.8	(0.163)
Increased	7.8	8.2	
Walking and standing			
Decreased	7.3	36.9	<0.001
No change	88.2	58.2	(0.341)
Increased	4.5	4.9	
Sedentary activity			
Decreased	3.0	4.0	<0.001
No change	88.6	65.8	(0.267)
Increased	8.4	30.2	
Sleep duration			
Became longer	5.5	27.2	<0.001
No change	88.9	65.2	(0.285)
Became shorter	5.6	7.6	
Waketime			
Became later	5.8	19.0	<0.001
No change	79.4	68.8	(0.189)
Became earlier	14.8	12.2	
Bedtime			
Became later	8.7	16.4	<0.001
No change	79.2	67.6	(0.133)
Became earlier	12.1	16.0	
Amount of drinking ^4^			
Increased	14.5	17.4	<0.001
No change	74.1	64.6	(0.108)
Decreased	11.3	18.0	
Amount of smoking ^5^			
Increased	16.2	26.0	0.006
No change	75.3	62.9	(0.133)
Decreased	8.5	11.1	
Frequency of defecation			
Increased	2.9	7.8	<0.001
No change	92.8	86.8	(0.106)
Decreased	4.3	5.4	
Frequency of main meals			
Increased	2.4	7.3	<0.001
No change	92.9	86.4	(0.115)
Decreased	4.7	6.3	
Frequency of snacking			
Increased	9.7	23.3	<0.001
No change	81.5	70.4	(0.176)
Decreased	8.8	6.4	
Body weight			
Increased	31.3	38.1	<0.001
No change	47.6	41.5	(0.072)
Decreased	21.1	20.5	

^1^ Changes in at-home hours during stay-at-home request period compared to those in the period before the COVID-19 pandemic. ^2^ Differences in distribution between groups were examined by chi-squared test. ^3^ Ranges from 0 to 1, where 1 indicates a strong association. ^4^ Excluding those who never drink. ^5^ Excluding those who never smoke.

## Data Availability

The data presented in this study are available on request from the corresponding author.

## Supplementary Materials

The following are available online at https://www.mdpi.com/article/10.3390/nu13082698/s1. Table S1: Changes in dietary intake during and after stay-at-home request period according to changes in at-home hours during stay-at-home request; Table S2: Lifestyle habits and body weight changes according to age group during and after stay-at-home request period among participants with extended at-home hours during stay-at-home request period (*n* = 3272).
