# Supplementary material for: Association between Lifestyle Changes and at-Home Hours during and after the State of Emergency Due to the COVID-19 Pandemic in Japan
Source: Nutrients. 2021 Aug 4;13(8):2698. doi: 10.3390/nu13082698 (PMC8398728; doi:10.3390/nu13082698)
Supplement: Supplementary file 1 [file nutrients-13-02698-s001.zip › nutrients-1316282-supplementary.pdf]

Table S1. Changes in food intake during and after stay-at-home request period according to changes in at-home hours during the stay-at-home request period.

	During Stay-at-Home Request Period		p-Value [2]	After Stay-at-Home Request Period		p-Value [2]
	Non extended At-Home Hours [1] (n = 6373)	Extended At-Home Hours [1] (n = 3272)		Non extended At-Home Hours [1] (n = 6373)	Extended At-Home Hours [1] (n = 3272)	
	%	%		%	%	
Foods increased in consumption compared to those before the COVID-19 pandemic [3]						
Cereals	12.3	25.2	<0.001	9.1	17.4	<0.001
Potatoes	1.5	4.0	<0.001	1.7	3.1	<0.001
Soy and soy products	5.0	11.1	<0.001	4.6	9.9	<0.001
Vegetables	5.5	13.1	<0.001	5.1	11.8	<0.001
Fruits	4.9	11.9	<0.001	5.2	10.8	<0.001
Seaweed	2.0	4.7	<0.001	1.9	4.2	<0.001
Fish and seafoods	2.8	6.3	<0.001	2.6	6.0	<0.001
Meat	5.0	9.8	<0.001	3.8	7.8	<0.001
Eggs and egg products	5.0	10.5	<0.001	3.7	8.2	<0.001
Milk and dairy products	7.1	16.7	<0.001	6.0	13.1	<0.001
Oily foods	3.5	7.9	<0.001	2.8	5.5	<0.001
Salty snacks and confectionary	10.8	26.8	<0.001	7.8	16.6	<0.001
Sugar-sweetened beverages	5.2	11.7	<0.001	4.1	8.5	<0.001
Others	0.5	1.4	<0.001	0.5	1.1	<0.001
Foods decreased in consumption compared to those before COVID-19 pandemic [3]						
Cereals	2.9	5.6	<0.001	3.7	6.5	<0.001
Potatoes	2.7	4.5	<0.001	2.5	3.8	<0.001
Soy and soy products	1.3	2.5	<0.001	1.7	2.6	0.002
Vegetables	2.9	5.8	<0.001	2.9	5.2	<0.001
Fruits	2.2	5.0	<0.001	1.9	3.7	<0.001
Seaweeds	1.6	3.1	<0.001	1.4	3.0	<0.001
Fish and seafoods	2.8	6.1	<0.001	2.5	5.0	<0.001
Meat	2.5	5.2	<0.001	2.4	4.5	<0.001
Eggs and egg products	0.9	2.0	<0.001	0.9	2.0	<0.001
Milk and milk products	1.6	2.3	0.009	1.4	2.8	<0.001
Oily foods	3.1	7.6	<0.001	2.8	7.1	<0.001
Salty snacks and confectionary	3.2	5.7	<0.001	3.5	7.4	<0.001
Sugar-sweetened beverages	2.8	6.1	<0.001	2.6	6.2	<0.001
Others	0.1	0.6	<0.001	0.2	0.3	0.598

[1] Changes in at-home hours during stay-at-home request period compared to those in the period before the COVID-19 pandemic. [2] Differences in distribution between groups were examined by chi-squared test. [3] Multiple answers were allowed.

Table S2. Lifestyle habits and body weight changes according to age group during and after stay-at-home request period among participants with extended at-home hours during stay-at-home request period (n = 3272).

	During Stay-at-Home Request Period					p-Value [1] (Cramer's V [2])	After Stay-at-Home Request Period					p-Value [1] (Cramer's V [2])
	20 s	30 s	40 s	50 s	≧60		20 s	30 s	40 s	50 s	≧60	
	%	%	%	%	%		%	%	%	%	%	
Number	437	499	534	458	1344		437	499	534	458	1344	
Total PA						<0.001						<0.001
Decreased	49.7	48.9	45.7	45.9	51.7	(0.093)	31.1	27.1	28.7	27.3	34.5	(0.117)
No change	35.9	39.1	42.5	46.1	43.0		48.7	61.5	59.4	64.6	59.2	
Increased	14.4	12.0	11.8	8.1	5.3		20.1	11.4	12.0	8.1	6.3	
Heavy physical work or strenuous exercise						<0.001						<0.001
Decreased	18.8	20.2	21.5	20.3	28.9	(0.130)	16.2	13.6	14.4	12.9	18.7	(0.107)
No change	65.7	66.9	67.2	73.6	67.4		68.0	76.2	76.2	79.9	76.9	
Increased	15.6	12.8	11.2	6.1	3.7		15.8	10.2	9.4	7.2	4.5	
Walking and standing						0.193						<0.001
Decreased	47.8	44.1	40.4	41.5	43.9	(0.041)	24.5	21.6	22.1	22.1	28.3	(0.080)
No change	47.6	51.3	54.1	55.5	52.4		66.6	73.7	71.9	75.1	68.1	
Increased	4.6	4.6	5.4	3.1	3.7		8.9	4.6	6.0	2.8	3.6	
Sedentary activity						<0.001						<0.001
Decreased	6.6	4.6	5.2	3.9	2.0	(0.076)	8.5	3.0	4.3	3.3	2.1	(0.110)
No change	56.3	62.7	60.1	62.7	57.2		68.2	79.6	79.4	81.0	71.9	
Increased	37.1	32.7	34.6	33.4	40.8		23.3	17.4	16.3	15.7	26.0	
Sleep duration						<0.001						<0.001
Became longer	50.1	37.9	39.0	27.3	19.0	(0.178)	33.0	21.2	19.7	14.2	13.5	(0.131)
No change	43.0	56.1	57.7	67.0	75.1		57.4	69.9	74.7	80.6	80.3	
Became shorter	6.9	6.0	3.4	5.7	5.9		9.6	8.8	5.6	5.2	6.2	
Waketime						<0.001						<0.001
Became later	41.6	31.3	33.0	21.6	15.0	(0.165)	24.3	14.4	17.4	10.0	9.7	(0.120)
No change	48.7	61.1	58.1	71.8	75.5		57.4	72.1	69.7	77.1	78.6	
Became earlier	9.6	7.6	9.0	6.6	9.5		18.3	13.4	12.9	12.9	11.8	
Bedtime						<0.001						<0.001
Became later	32.3	21.8	18.2	16.6	13.5	(0.139)	22.2	12.2	14.0	12.7	10.6	(0.096)
No change	50.3	59.7	59.7	69.9	73.6		60.0	71.1	69.7	74.7	77.1	
Became earlier	17.4	18.4	22.1	13.5	12.9		17.8	16.6	16.3	12.7	12.4	
Amount of drinking [3]						<0.001						<0.001
Increased	22.6	29.7	25.2	21.1	16.1	(0.164)	18.6	15.8	17.3	15.2	15.5	(0.116)
No change	49.2	54.1	59.9	70.3	74.6		54.3	62.6	68.4	72.7	73.3	
Decreased	28.1	16.2	15.0	8.6	9.3		27.1	21.6	14.3	12.1	11.2	

Amount of smoking [4]						0.006						0.129
Increased	35.2	35.2	34.4	20.4	22.9	(0.135)	31.0	26.4	24.2	18.4	17.2	(0.104)
No change	46.5	52.7	57.8	68.9	68.8		53.5	63.7	68.0	70.9	73.4	
Decreased	18.3	12.1	7.8	10.7	8.3		15.5	9.9	7.8	10.7	9.4	
Frequency of defecation						<0.001						<0.001
Increased	13.7	9.4	6.9	3.9	4.4	(0.097)	11.0	6.8	5.4	4.8	4.2	(0.078)
No change	80.5	86.2	88.2	91.5	91.7		82.4	87.0	89.0	90.8	91.9	
Decreased	5.7	4.4	4.9	4.6	3.9		6.6	6.2	5.6	4.4	3.9	
Frequency of main meals						<0.001						<0.001
Increased	17.4	16.4	12.7	9.2	6.1	(0.155)	11.0	7.4	4.1	3.9	3.6	(0.129)
No change	71.2	78.4	82.4	88.9	91.7		76.4	85.8	89.5	91.9	93.2	
Decreased	11.4	5.2	4.9	2.0	2.2		12.6	6.8	6.4	4.1	3.1	
Frequency of snacking						<0.001						0.001
Increased	38.2	36.1	33.7	28.8	26.6	(0.080)	23.6	18.6	16.7	16.6	16.4	(0.062)
No change	57.0	59.3	61.8	68.6	70.2		66.8	72.5	75.3	76.2	77.8	
Decreased	4.8	4.6	4.5	2.6	3.1		9.6	8.8	8.1	7.2	5.9	
Body weight						<0.001						<0.001
Increased	32.5	39.7	46.4	44.1	38.2	(0.097)	25.9	34.1	38.2	38.9	36.5	(0.092)
No change	45.5	42.7	40.4	44.3	50.7		43.0	42.3	40.3	43.2	46.6	
Decreased	22.0	17.6	13.1	11.6	11.0		31.1	23.6	21.5	17.9	16.9	

[1] Differences in distribution between groups were examined by chi-squared test. [2] Ranges from 0 to 1, where 1 indicates a strong association. [3] Excluding those who never drink. [4] Excluding those who never smoke.
